# Utility of total lymphocyte count as a surrogate marker for CD4 counts in HIV-1 infected children in Kenya

**DOI:** 10.1186/1471-2334-11-259

**Published:** 2011-09-30

**Authors:** Nyawira Githinji, Elizabeth Maleche-Obimbo, Moses Nderitu, Dalton C Wamalwa, Dorothy Mbori-Ngacha

**Affiliations:** 1 Department of Pediatrics and Child Health, University of Nairobi, Nairobi, Kenya; 2 Kenya Medical Research Institute-Wellcome Trust Research Program, Nairobi, Kenya

**Keywords:** Total Lymphocyte Count, TLC, CD4, HIV, Children, surrogate marker

## Abstract

**Background:**

In resource-limited settings, such as Kenya, access to CD4 testing is limited. Therefore, evaluation of less expensive laboratory diagnostics is urgently needed to diagnose immuno-suppression in children.

**Objectives:**

To evaluate utility of total lymphocyte count (TLC) as surrogate marker for CD4 count in HIV-infected children.

**Methods:**

This was a hospital based retrospective study conducted in three HIV clinics in Kisumu and Nairobi in Kenya. TLC, CD4 count and CD4 percent data were abstracted from hospital records of 487 antiretroviral-naïve HIV-infected children aged 1 month - 12 years.

**Results:**

TLC and CD4 count were positively correlated (r = 0.66, p < 0.001) with highest correlation seen in children with severe immuno-suppression (r = 0.72, p < 0.001) and children >59 months of age (r = 0.68, p < 0.001). Children were considered to have severe immuno-suppression if they met the following WHO set CD4 count thresholds: age below 12 months (CD4 counts < 1500 cells/mm^3^), age 12-35 months (CD4 count < 750 cells/mm3), age 36-59 months (CD4 count < 350 cells/mm^3^, and age above 59 months (CD4 count < 200 cells/mm^3^). WHO recommended TLC threshold values for severe immuno-suppression of 4000, 3000, 2500 and 2000 cells/mm^3 ^for age categories <12, 12-35, 36-59 and >59 months had low sensitivity of 25%, 23%, 33% and 62% respectively in predicting severe immuno-suppression using CD4 count as gold standard. Raising TLC thresholds to 7000, 6000, 4500 and 3000 cells/mm^3 ^for each of the stated age categories increased sensitivity to 71%, 64%, 56% and 86%, with positive predictive values of 85%, 61%, 37%, 68% respectively but reduced specificity to 73%, 62%, 54% and 68% with negative predictive values of 54%, 65%, 71% and 87% respectively.

**Conclusion:**

TLC is positively correlated with absolute CD4 count in children but current WHO age-specific thresholds had low sensitivity to identify severely immunosuppressed Kenyan children. Sensitivity and therefore utility of TLC to identify immuno-suppressed children may be improved by raising the TLC cut off levels across the various age categories.

## Background

There are an estimated 200,000 HIV-1 infected children in Kenya the majority of whom acquired the infection perinatally [1,2]. Without treatment, the mortality of these children in Kenya and similar resource-poor settings approaches 50% by the age of 2 years, with most deaths attributable to infectious illnesses and failure to thrive [3-6]. Recent scale-up of highly active antiretroviral therapy (HAART) has resulted in improved survival, however less than 30% of eligible children are currently receiving HAART [7-10]. Factors that undermine further expansion of pediatric HAART coverage in the Kenyan context include late diagnosis, lack of health personnel trained in antiretroviral therapy (ART) delivery and limited laboratory infrastructure for CD4 testing [2]. The critical role of CD4 cell count/percent in predicting clinical progression of pediatric HIV-1 is well described [11-13]. The diagnostic work-up of HIV-1 infected children is considered incomplete without review of CD4 results despite the fact that this test is not routinely available in most rural Kenyan settings that bear the greatest burden of pediatric HIV. There are an estimated 100 machines for CD4 testing (FACSCount ^® ^or FACSCalibre ^®^) in Kenya of which only 35 are located in public health facilities which serve the majority of HIV-1 infected children, while the remainder are found in large private hospitals and clinics, largely in urban settings. The cost of performing a CD4 count in Kenya is estimated at US$12 which is at least 4 times higher than that of a total lymphocyte count. In 2006 the World Health Organization (WHO) recommended the use of total lymphocyte count (TLC) as a guide for initiating ART in children with WHO clinical stage 2 who are aged 8 years and below in settings where CD4 counts are not available [14]. In adult studies, correlation between the TLC and CD4 counts ranged from 0.64 to 0.78, and appeared to be stronger for patients with advanced disease [15-18].

The sensitivity of the recommended TLC levels of <1200 cells/mm^3 ^for predicting CD4 counts below 200 cells/mm^3 ^in adult studies has been however found to vary widely from 38% to 75% while the positive predictive value has ranged between 64% to 88%[16-19]. A sensitivity of 75% means that the recommended TLC cut off will only detect three quarters of those with true CD4-defined immuno-suppression and miss one quarter. On the other hand a positive predictive value of 88% implies that among children found to have immuno-suppression by a given TLC cut-off, 88% will meet the CD4 criteria for immuno-suppression [20]. Given the aggressive nature of pediatric HIV, any diagnostic test employed must have very high sensitivity since missing a diagnosis of severe immuno-suppression would result in increased mortality [3]. On the other hand low positive predictive value would lead to misclassification with children who otherwise have good immunity being categorized as severely immunosuppressed and inadvertently started on HAART, thus increasing costs and risk of toxicity. A meta-analysis by Dunn on a large group of children in US and Europe found TLC <2500 cells/mm^3 ^or CD4 percent <20% to be associated with high mortality [21].

As part of the Kenya's efforts to meet the recently published PEPAR II goals, an estimated 100,000 children need to be newly initiated on HAART before 2013 [22]. In order to realize this goal, new treatment centers need to be established and some of these may not immediately have access to routine CD4 testing. Hence it is pertinent" to validate the use of TLC as surrogate for CD4 counts in local settings. This is especially critical since most existing data are largely drawn from the US and Western Europe and may not be directly applicable to local settings. Furthermore there is need for region specific validation of CD4 and TLC counts changes both "Pre-HAART" and on HAART. Our study was designed to determine the diagnostic utility of TLC as a surrogate for CD4 counts and correlation between TLC and CD4 counts in Kenya children attending HIV clinics in three sites.

## Methods

This was a retrospective hospital based cross-sectional survey, carried out at three hospitals in Kenya; Kenyatta National Referral Hospital (KNH) in Nairobi, Mbagathi District Hospital (MDH) in Nairobi, and New Nyanza Provincial General Hospital (NNPGH) in Kisumu, western Kenya. All HIV infected children aged between one month and twelve years on follow-up in the comprehensive HIV care clinics (CCC) at the three hospitals who had medical records on CD4 count and total lymphocyte at enrollment into care prior to initiation of ART were eligible for inclusion in the study.

The medical records of children receiving care between January 2004 and July 2006 were reviewed. "Evidence of HIV status was sought, specifically, by documentation of a positive HIV antibody test for children above 18 months, or positive HIV PCR test result for children below 18 months. For eligible children, data on age, sex, WHO clinical stage, absolute CD4 count and TLC data prior to ART initiation was abstracted from medical records. Any child missing any of these data was excluded. Children were consecutively enrolled from each site until the desired sample size was achieved. We excluded children under the age of one month due to uncertainty of HIV diagnosis in this group.

Approval to conduct the study was sought from the Ethical Review Committee of KNH which is the teaching hospital of the University of Nairobi, as well as from the administration of the Mbagathi and New Nyanza Hospitals. Confidentiality was maintained for all abstracted patient records which were identified only by number stored in a secure computer.

### Statistical Methods

Data was analyzed using STATA version 9.2. Correlation between all pairs of TLC, CD4 cell count, and TLC and CD4% (all as continuous variables) were determined using Spearman's correlation coefficient. Children were then categorized into four age groups <12, 12-35, 36-59 and >59 months. We also categorized children by presence or absence of severe immuno-suppression based on WHO 2006 age specific immunologic categorizations as follows: age <12 months CD4 < versus ≥ 1500 cells/mm^3^, 12-35 months CD4 < versus ≥ 750 cells/mm^3^, 36-59 months CD4 < versus ≥ 350 cells/mm^3 ^and >59 months CD4 < versus ≥ 200 cells/mm^3^. Similarly severe immune-suppression by CD4 percentage was defined as CD4% <25%, <20%, <15% and <15% for ages <12 months, 12-35, 36-59, and >59 months respectively [14].

TLC was categorized using WHO age-specific TLC thresholds defining presence or absence of immuno-suppression stratified by the same age categories as follows: < versus ≥ 4000 cells/mm^3^, 3000 cells/mm^3^, 2500 and 2000 cells/mm^3 ^respectively [14]. The sensitivity, specificity, positive predictive value (PPV) negative predictive values (NPV) of the WHO TLC cut-offs in predicting severe immune-suppression as defined by WHO CD4 thresholds were determined. Data was analyzed to determine the optimal TLC cut-offs that provide highest sensitivity and specificity for predicting severe immune-suppression.

## Results

We identified 487 ART naive HIV infected children who had complete records, including 125 children from NNPGH, 186 from MDH and 176 from KNH. Two hundred and twenty three (46%) were female and the median age was 36 months (IQR 18-68, Table 1).

**Table 1:** Descriptive Characteristics of Study Population (n = 487)

Characteristic	Frequency (%) or Median (IQR)
Hospital	
New Nyanza Provincial Hospital	125 (25.7)
Mbagathi District Hospital	186 (38.2)
Kenyatta National Hospital	176 (36.1)

Female	223 (46)

Age in months	36 (18-68)

Age group	
<12 months	82 (17)
12-35 months	129 (26)
36-59 months	118 (24)
>59 months	158 (32)

WHO Clinical Stage	
1 and 2	96 (19.7)
3 and 4	390 (80.2)

### Clinical Parameters and CD4 Profile

Prior to starting ART 95 (20%) children had WHO clinical stage 1 or 2 disease, while 392 (80%) had stage 3 or 4 disease (Table 1). The median CD4 count was 537 cells/mm^3 ^(IQR 216-992), median CD4 percentage 11.6% (IQR 5.7-19), and median TLC (TLC) was 4500 cells/mm^3 ^(IQR 2700-6655). The median CD4 count decreased with rising age, and was 1060, 777, 565 and 226 cells/mm^3 ^in children of age <12, 12-35, 36-59 and >59 months respectively. The corresponding median CD4% for children in the four age categories in ascending order was 17.5%, 12.3%, 11.9%, and 7.4%. The median TLC was 6895, 6000, 4513 and 2577 cells/mm^3 ^among children of the four age groups (Table 2). We categorized children as either severely immunosuppressed or not based on age-specific thresholds of CD4 count, CD4%, and TLC based on WHO 2006 classifications (Table 2).

**Table 2:** CD4 counts and Percent and Total Lymphocyte Count by Age Category

Age group in months	CD4 count (cells/mm^3^) Median(range)	CD4 percentage Median(range)	TLC (cells/mm^3^) Median(range)	No. of children
<12	1060 (604-1590)	17.5 (10.5-23.6)	6895 (4875-8670))	82
12-35	777 (400-1123)	12.3 (7.2-18)	6000 (3770-8035)	129
36-59	565 (250-880)	11.9 (5-8-21.9)	4513 (3289-5511)	118
>59	226 (35-430)	7.4 (1.6-14)	2577 (1675-4100)	158
ALL	537 (216-992)	11.6 ((5.7-190)	4500 (2700-6655)	487

### Correlation between CD4 Counts and Total Lymphocyte Counts

Correlation between absolute CD4 counts and the TLC for the whole study population was 0.66 (Spearman correlation coefficient). In the individual sites correlation was as follows: KNH (0.76), MDH (0.55), and NNPGH (0.54). Correlation between CD4 counts and TLC was then explored among different age groups, and stratified by presence or absence of severe immuno-suppression. Correlation among children <12 months was 0.54, for 12-35 months it was 0.44, for 36-59 months, 0.38, and for >59 months correlation was 0.68. The strongest correlation was seen in the oldest group of children (>59 months). Correlation between CD4 count and TLC among children who were severely immunosuppressed was 0.72 which was higher than their counterparts without severe immuno-suppression correlation 0.63. Overall correlation between TLC and CD4% was weak (r = 0.06).

### Diagnostic Utility of TLC to identify Severely Immuno-suppressed Children

To test the utility of TLC as a surrogate marker for CD4 count, sensitivity, specificity, PPV and NPV of TLC was determined using WHO 2006 recommended cut off values for absolute CD4 count as the gold standard. Table 3 shows the proportion of children classified according to presence of severe immuno-suppression as defined by both CD4 count and TLC and stratified by age while table 4 shows the same with additional stratification by WHO clinical stage. The sensitivity of WHO recommended age-specific TLC thresholds to identify severely immune-suppressed children was low, ranging from 23 to 62% while specificity was high ranging from 83% to 100%. PPV was high ranging from 68% to 100% while NPV ranged from 37% to 74% (Table 5).

**Table 3:** Proportion of Children Immuno-suppressed by Age Category

Age group in months	Severely depressed CD4 count Frequency (%)	Severely depressed CD4 percentage Frequency (%)	Depressed TLC Frequency (%)	No. of Children
<12	56 (68.3)	62 (48.1)	13 (15.9)	82
12-35	62 (48.1)	102 (79.1)	15 (12.4)	129
36-59	39 (33.3)	75 (63.6)	19 (16.1)	118
>59	69 (43.7)	127 (80.4)	58 (36.7)	158

ALL	226 (46.2)	371 (76.2)	105(21.6)	487

**Table 4:** Proportion of Children Immuno-suppressed by Age Category stratified by WHO clinical stage.

WHO Clinical Stage	Age group in months	Severely depressed CD4 count Frequency (%)	Depressed TLC Frequency (%)	No. of Children
Stage 1-2	<12	12 (60)	1 (5)	20
	12-35	9 *(35)	1 (3.8)	26
	36-59	4 (13)	5 (16.1)	31
	>59	5 (26)	7 (36.8)	19
	All	30 (31)	14 (14.6)	96

Stage 3-4	<12	44 (71)	12 (19.4)	62
	12-35	52 (51)	14 (13.7)	102
	36-59	35 (40)	14 (16.1)	87
	>59	64 (46)	51 (36.7)	139
	All	195 (50)	91 (23.3)	390

ALL		225 (46)	105 (21.6)	486*

*One child in this age category did not have WHO clinical stage excluded from this table

**Table 5:** Sensitivity, Specificity, PPV and NPV of Current WHO TLC thresholds and Proposed new raised TLC thresholds to Identify Severely Immuno-suppressed Children

Age group in months	Current WHO TLC threshold (cells/mm^3^)	Sensitivity	Specificity	PPV^a^	NPV^b^	No. of children
<12	4000	25	100	100	37	82
12-35	3000	23	98	93	58	129
36-59	2500	33	92	68	74	118
>59	2000	62	83	74	74	158
ALL		36^c^	93^c^	84^c^	61^c^	487

Age group in months	Proposed Optimal TLC	Sensitivity	Specificity	PPV	NPV	No. of Children

<12	7000	71	73	85	54	82
12-35	6000	65	62	61	65	129
36-59	4500	60	54	37	71	118
>59	3000	86	68	68	87	158
ALL		71	64	63	69	487

^a^PPV = positive predictive value ^b^NPV = negative predictive value ^c^Mean value for the four age groups

### Raising Cut off Values of TLC to Improve Diagnostic Utility

**Figure 1:**
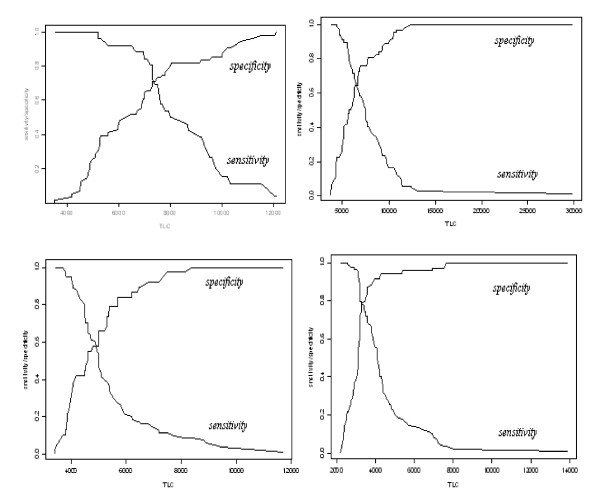
**Optimal sensitivity and specificity for various TLC cut-offs by Age Group**. Sensitivity and specificity of TLC to detect severe immune-suppression was plotted against various total lymphocyte count (cells/mm^3^) cut-offs for four different age groups. a) Below 12 months b) 12-35 months c) 36-59 months and d) 60 months and above

Since the recommended WHO TLC cut offs for identifying severe immuno-suppression in all age categories had low sensitivity, we explored whether raising the TLC cut offs would improve the sensitivity without unduly compromising the specificity. As an alternative to receiver operating characteristic curve for simultaneously evaluating how sensitivity and specificity vary as the cutoff point is changed, we plotted sensitivity and specificity against various TLC cut off points. The plots for different age groups were used to determine optimal cut off points which are defined as the TLC value that yields the highest sensitivity while maintaining acceptable specificity (Figure 1). For age category <12 months, raising the TLC cut off from WHO 4000 cells/mm^3 ^to 7000 cells/mm^3 ^improved the sensitivity from 25% to 71%, but decreased specificity from 100% to 73% (Table 4). Similarly for age group 12-35 months, raising the TLC cut off value from 3000 cells/mm^3 ^to 6000 cells/mm^3 ^improved the sensitivity from 23% to 65% accompanied by a decreased specificity from 98% to 62%. For age category 36-59 months, raising the TLC cut off value from 2500 cells/mm^3 ^to 4500 cells/mm^3 ^would improve the sensitivity from 33% to approximately 60%. For age category >59 months, raising the TLC cut off from 2000 cells/mm^3 ^to 3000 cells/mm^3 ^would improve the sensitivity from 62% to 86% but lead to a loss in specificity from 83% to 68%. A mean value across age groups was computed for each variable at the current WHO TLC threshold, and at new proposed threshold. The mean sensitivity improved considerably from 36% to 71%, mean specificity decreased from 93% to 64%, mean PPV dropped from 84% to 63%, mean NPV improved from 61% to 69% (Table 5).

## Discussion

Several studies in adults and a few in children have suggested that TLC may be used as surrogates of CD4+ T cell count in HIV infected patients [17,18,22,23]. Our study was designed to evaluate the utility of TLC to identify immuno-suppression in HIV infected children from three pediatric HIV care centers in Kenya. In our study population, TLC was positively correlated with CD4 count (overall r = 0.66), with stronger correlation among children with severely depressed CD4 counts. We found the WHO 2006 TLC thresholds defining immuno-suppression to have low sensitivity but high specificity for detecting severely depressed CD4 count in these Kenyan children, and sensitivity was lowest among young children under 5 years (<34%). In contrast to the CD4 count, there was very poor correlation between TLC and CD4% (r = 0.06) similar to weak correlation (r = 0.01) reported by Musoke in Ugandan children [24].

The WHO recommendations for use of TLC as surrogate marker for CD4 count for decision in initiating HAART in children is based upon the premise that TLC is easily available and easy to perform relative to the CD4 assays. As a good surrogate marker the TLC cut-off values should be highly sensitive in identifying children of low CD4 count requiring HAART. Stated differently, a good tool should not miss immuno-suppressed children who are at highest risk of mortality. In our study population, between 67 and 75% of the children aged below 5 years who require ART would have been missed by the WHO TLC thresholds used suggesting low utility in HIV program settings.

In order to improve the utility of TLC we explored raising the cut-off values to levels higher than those recommended by the WHO 2006 guidelines. We used the point of maximal sensitivity with least compromise in specificity as the new optimal TLC cutoff point. We thus selected increased TLC thresholds that would give optimized combination of good sensitivity, with acceptable specificity and PPV. This resulted in improved mean sensitivity of the TLC from 36% to 71% for detection of severe immuno-suppression in our population, while retaining acceptable specificity (mean 68%) and PPV (mean 63%). Examining the effect of raising TLC threshold more closely, in the age category <12 months, the proposed raised TLC threshold of 7000 cells/mm^3 ^yielded improved sensitivity of 71% (previously 25%), meaning that new threshold would identify 7 of 10 immuno-suppressed children needing ART compared to 2 of 10 identified by the previous threshold of 4000 cells/mm^3^. Given that HIV is uniformly fatal in the absence of interventions, improved sensitivities for the raised TLC threshold favors the use of TLC as a screening tool for severe immuno-suppression in settings where CD4 monitoring is not easily accessible.

On the other hand, further increasing TLC thresholds beyond the optimized levels we selected based on our cohort data would grossly decrease specificity and PPV for relatively little incremental gain in sensitivity. This would result in unacceptably high numbers of children who are actually not severely immune-depleted being misclassified and started on ART earlier than they require, exposing them unnecessarily to the problems associated with ART including adverse drug effects, development of antiretroviral drug resistance well as increasing the cost of running programs purchase of expensive medications.

Considering that the clinical staging of disease is also employed together with the immunological criteria in order to initiate HAART, in this study we recommend raising the TLC cut-offs from the current WHO recommended TLC cut-off values. Raising TLC cut off values to improve sensitivity and enable TLC to be used as a better screening tool has been previously proposed by adult studies in Uganda and Kenya [17,18]. A Ugandan study in adults found TLC values of 2100 cells/mm^3 ^to best predict CD4 < 200 cells/mm^3 ^(sensitivity 83%, specificity 77%, PPV 92%, NPV 57%) which was superior to a cut-off value of 1200 [19]. In Kenyan adults, raising the TLC cut off value from the recommended 1200 cells/mm3 to 1900 cells/mm3 resulted in improved diagnostic utility for predicting CD4 < 200 cells/mm^3 ^(sensitivity 81%, specificity 90%, PPV 90%, NPV 80%) [17]. The higher cut off value for TLC required to improve the utility of this tool in our setting may be due to higher burden of background infectious disease. WHO recommended cut-off TLC values were largely driven by available data from the United States. It has been previously shown that normal values of T cell subset among African children may differ from those of other populations. A study in Guinea Bissau found healthy children under the age of 2 years to have lower CD4% and CD4/CD8 ratios and higher CD8% than their counterparts from developed countries [25].

Mahajan et al explored the utility of changes in TLC as a surrogate for changes in CD4 counts following HAART initiation among HIV-infected adults in India [26]. The main strength of this study was the multiple longitudinal TLC measurements with corresponding CD4 counts that allowed a comparison of changes as opposed to single value as is the case in our study. The study showed that an increase in TLC reliably predicts a corresponding rise in CD4 count but a falling TLC did not similarly predict a drop in CD4 count (98% versus 63% positive and negative predictive values respectively. The study also noted substantial individual variation making it more difficult for a single TLC measurement to predict the actual quantitative CD4 change further highlighting the importance of multiple measurements [26].

The poor correlation between TLC and CD4% in children in our study is of concern given that CD4% is widely used in children to predict disease progression and is included in WHO guidelines for initiating HAART[14]. Moreover, CD4% has been found to be an important prognostic marker even post HAART initiation in a large cohort of HIV Zambian infected children [27]. It is notable that in the Zambian study, low CD4% was associated with mortality mainly among children < 18 months but not in those aged 18-59 months. Similarly in a cohort of HAART-treated HIV infected children in Kwazulu Natal, CD4% was not predictor of mortality underscoring the fact that TLC may still be relevant despite lacking direct association with CD4% [28].

An important group of HIV-infected children are those co-infected with tuberculosis as well as other conditions associated with leukocyte changes independent of HIV. Although no pediatric literature was found on this subject, among adults, Martin et al found strong correlation (R = 0.7) between TLC and CD4 count both at baseline and after 1 month of TB treatment in HIV-TB co-infected South African adults [29]. In this study TLC of between 1300-1500/ml was highly predictive of CD4 count< 200/ul.

In a broad context of expanding HIV treatment in settings with limited laboratory recourses, the DART trial, a randomized non-inferiority trial comparing clinically driven monitoring (CDM) verses laboratory and clinical monitoring (LCM) found clinically 5 year survival rates of 87% verses 90% in CDM and LCM arms respectively [30]. While 12-weekly CD4 monitoring did not impact disease progression in the first 2 years of ART, after 2 years a small but significant increase in clinical disease progression was found in favour of the LCM. This clinical progression may be attributed to delay in switching to 2^nd ^line ART in the CDM with potential higher resistance. Thus whereas findings of the DART trial highlight the importance of CD4 testing in patients it remains crucial to similarly evaluate the role of TLC as surrogate for CD4 in HAART-treated children.

## Conclusions

We conclude that in these Kenyan children TLC was positively correlated with absolute CD4 count, but current WHO age-specific TLC thresholds had low sensitivity to identify those who were severely immuno-suppressed. Raising the TLC cut-off for all age groups considerably improved the sensitivity of TLC, retaining acceptable specificity and PPV, suggesting that at higher thresholds TLC has good utility as a tool to identify severely immune-depleted children requiring ART in settings where CD4 testing is not readily available. Limitations of our study include the modest sample size and fact that we only obtained single measurements of TLC and CD4. Our findings suggest that rather than shelve the use of TLC due to concerns of diagnostic utility, further research is needed to define optimum cut off values and should preferably be longitudinal with a much larger sample size. Considering the significantly higher cost of performing a CD4 count relative to TLC, and the limited availability of FACSCOUNT machines in Kenya, efforts that will lead to adoption of TLC will remove a major barrier to HAART initiation for HIV infected children, avert numerous deaths and help the country meet the targets of PEPfAR II.

## Competing interests

The authors declare that they have no competing interests.

## Authors' contributions

NG participated in the design, data collection, and drafting of the manuscript. EMO conceived the study, and participated in its design and drafting of the manuscript. MN performed the statistical analysis. DW participated in statistical analysis and manuscript review. DMN participated in the drafting of the manuscript. All authors read and approved the final manuscript.

## Pre-publication history

The pre-publication history for this paper can be accessed here:

http://www.biomedcentral.com/1471-2334/11/259/prepub
